# Balanced crystalloid solutions versus normal saline in intensive care units: a systematic review and meta-analysis

**DOI:** 10.1007/s11255-023-03570-9

**Published:** 2023-04-05

**Authors:** Puze Wang, Yin Huang, Jin Li, Dehong Cao, Bo Chen, Zeyu Chen, Jinze Li, Ruyi Wang, Liangren Liu

**Affiliations:** 1 Department of Urology, West China Hospital of Medicine, Chengdu, China; 2 Department of Urology, Hospital of Chengdu University, Chengdu, China

**Keywords:** Balanced crystalloid solutions, Intensive care units, Mortality, Renal-related outcomes, Meta-analysis

## Abstract

**Background:**

Intravenous fluid therapy is important for pediatric and adult patients in intensive care units (ICUs). However, medical professionals continue to struggle to determine the most appropriate fluids to obtain the best possible outcomes for each patient.

**Objective:**

We conducted a meta-analysis involving cohort studies and randomized controlled trials (RCTs) to compare the influence of balanced crystalloid solutions and normal saline among patients in ICUs.

**Patients and methods:**

Studies that compared balanced crystalloid solutions and saline in ICU patients from databases including PubMed, Embase, Web of Science, and Cochrane Library were systematically searched up to July 25, 2022. The primary outcomes were mortality and renal-related outcomes, which included major adverse kidney events within 30 days (MAKE30), acute kidney injury (AKI), new receipt of renal replacement therapy (RRT), maximum creatinine increasing, maximum creatinine level, and final creatinine level ≥ 200% of baseline. Service utilization including length of hospital stay, ICU stay, ICU-free days and ventilator-free days were also reported.

**Results:**

A total of 13 studies (10 RCTs and 3 cohort studies) involving 38,798 patients in ICUs met the selection criteria. Our analysis revealed that each subgroup had no significant difference in mortality outcomes among ICU patients between balanced crystalloid solutions and normal saline. A significant difference was detected between the adult groups (odds ratio [OR], 0.92; 95% confidence interval [CI], [0.86, 1.00]; *p* = 0.04) indicating that the AKI in the balanced crystalloid solutions group was lower than that in the normal saline group. Other renal-related outcomes, such as MAKE30, RRT, maximum creatinine increasing, maximum creatinine level, and final creatinine level ≥ 200% of baseline showed no significant difference between the two groups. Regarding secondary outcomes, the balanced crystalloid solution group had a longer ICU stay time (WMD, 0.02; 95% CI, [0.01, 0.03]; *p* = 0.0004 and *I*^2^ = 0%; *p* = 0.96) than the normal saline group among adult patients. Furthermore, children treated with balanced crystalloid solution had a shorter hospital stay time (WMD, − 1.10; 95% CI, [− 2.10, − 0.10]; *p* = 0.03 and *I*^2^ = 17%; *p* = 0.30) than those treated with saline.

**Conclusions:**

Compared with saline, balanced crystalloid solutions could not reduce the risk of mortality and renal-related outcomes, including MAKE30, RRT, maximum creatinine increasing, maximum creatinine level, and final creatinine level ≥ 200% of baseline, but the solutions may reduce total AKI incidence among adult patients in ICUs. For service utilization outcomes, balanced crystalloid solutions were associated with a longer length of ICU stay in the adult group and shorter length of hospital stay in the pediatric group.

**Supplementary Information:**

The online version contains supplementary material available at 10.1007/s11255-023-03570-9.

## Introduction

Compared with common hospital wards, the intensive care unit (ICU) is a specialized unit with more necessary support equipment and rescue resources, as it must provide life care and support to critically ill patients, stop or slow down organ failure processes, and ultimately reduce the risk of death [1]. For pediatric and adult patients in critical situations, fluid therapy is essential in resuscitation and maintenance [2], and proper intravenous fluid therapy can maintain vascular volume and improve organ perfusion [3, 4]. Normal 0.9% saline and balanced crystalloid solutions are common medical intravenous fluids that are widely used in ICUs and other hospital departments.

Normal saline is an isotonic fluid containing a high concentration of chloride ions, compared with plasma, while balanced crystalloid solutions have a similar chloride concentration to that of plasma [5]. A growing body of evidence suggests that high chloride concentrations in the circulatory system may increase the risk of adverse outcomes in both adults and children [6, 7]. The possible reason for these outcomes is that compared with balanced crystalloid fluids, 0.9% saline is more likely to lead to an internal electrolyte imbalance that may cause hyperchlorinated metabolic acidosis, a reduction in the glomerular filtration rate and fluid overload in the body [8–10]. Previous studies have shown that normal saline is associated with a higher incidence of renal dysfunction and overall mortality than balanced crystalloid solutions [11].

In recent years, a number of studies have evaluated balanced multielectrolyte solutions as a replacement for normal saline for intravenous fluid resuscitation in patients supported in ICUs. Hayes [12] showed that balanced crystalloid fluids were more suitable for critically ill children than saline and could also significantly reduce overall mortality and renal-injury events in adults. Nevertheless, some literature has reported that balanced crystalloid solutions did not effectively improve prognosis in ICU patients, compared with normal saline [5, 13]. Because of physiological differences, fluid therapy in children can be extremely different from that in adults. In addition, various intravenous fluid protocols remain are based on the cause of admission to the ICU. To provide clear guidance on intravenous fluid therapy among patients in ICUs, further research is needed to compare balanced crystalloid solutions and normal saline. Therefore, our systematic review and meta-analysis was conducted to investigate related outcomes.

## Materials and methods

### Literature research

Our prospective study followed the PRISMA (Preferred Reporting Items for Systematic Reviews and Meta-Analysis 2020 statement, Supplement Table 1) and PROSPERO register (Number: CRD42022347375). Databases, including PubMed, Embase, Web of Science (WOS) and Cochrane library, were systematically searched for primary literature that compared saline and balanced crystalloid fluids among ICU patients up to February, 2023. The following items were used in our search: “Balanced crystalloid solutions,” “saline,” and “intensive care units” (the specific search strategy is shown in Supplement Table 2).

### Identification of eligible studies

The following studies were considered eligible: (1) study design was randomized controlled trials (RCT) or cohort studies; (2) studies were conducted on ICU patients; (3) studies comparing balanced crystalloid and normal saline; (4) at least one mortality outcome (28-day mortality; 30-day mortality; 60-day mortality; 90-day mortality; or hospital mortality), renal function (major adverse kidney events within 30 days (MAKE30), acute kidney injury (AKI), new receipt of renal replacement therapy(RRT), maximum creatinine increasing, maximum creatinine level and final creatinine level ≥ 200% of baseline) or service utilization outcomes (length of hospital stay, ICU stay, ICU-free days, and ventilator-free days) was evaluated; and (5) sufficient data available to calculate odds ratio (OR) or weighted mean difference (WMD). Reviews, letters, editorial comments, case reports, unpublished articles, study protocols, and non-English articles were excluded. For different primary studies enrolling participants from the same database, we only included the study with the largest sample size if they had same experimental timing and inclusion criteria. In contrast, the literature with dissimilar study periods and inclusion standards were also included.

### Data extraction

Two investigators (Puze Wang and Yin Huang) performed data extractions independently, and a third investigator (Liangren Liu) made a final decision if there were any disagreements. Data for the following aspects were recorded: (1) basic literature information: first author, publication year, study period, country of study, study design, sample size, type of balanced crystalloids, center numbers; (2) demographic data: age, sex, comorbidities, renal functional indicators (creatinine, blood urea nitrogen, and estimate glomerular filtration rate, [EGFR]) and critical illness index (SOFA score and APACHE II score); (3) mortality outcomes: 28-day, 30-day, 60-day, 90-day, and hospital mortality; (4) renal-related outcomes: MAKE30, AKI, RRT, maximum creatinine increasing, maximum creatinine level, and final creatinine level ≥ 200% of baseline; (5) service utilization outcomes: length of hospital stay, ICU stay, ICU-free days, and ventilator-free days. The meaning of maximum creatinine increasing was described as “a standard that measuring the degree of change in kidney function” because changes in the creatinine concentration reflect the quality of kidney function indirectly. In addition, AKI was qualitatively defined as “abrupt and sustained decrease in glomerular filtration, urine output, or both” [14], and if possible, we extracted data of AKI, respectively, according to the KDIGO criteria [15].

If studies reported continuous variables such as the median with range or interquartile range, we used the validated mathematical method to obtain a concrete mean ± standard deviation [16, 17].

### Quality assessment

The Newcastle–Ottawa Scale (NOS) and Cochrane Risk of Bias Assessment Tool (CRBAT) were utilized to evaluate the quality of the included studies [18, 19]. Studies scoring 7–9 points were considered to be high quality [20]. In addition, the evidence level for every study was also assessed according to the Oxford Centre for Evidence-Based Medicine Levels of Evidence Working Group [21]. Evaluations were launched by the same two investigators independently, and any discrepancies were resolved through discussion. All related information are shown in Supplement Table 3.

### Statistical analysis and publication bias

Review Manager version 5.4 (Cochrane Collaboration, Oxford, UK) was used to perform evidence synthesis. We compared dichotomous and continuous variables applied for OR and WMD, and used 95% confidential intervals [CIs] to report on all metrics; the Chi-squared (*χ*^2^) test and inconsistency index (*I*^2^) were used to assess any potential heterogeneities in our included studies. An *χ*^2^ *P* value < 0.05 or *I*^2^ > 50% indicated that significant heterogeneity existed. A random-effect model was used to estimate the combined WMD or OR was used when significant heterogeneity was detected (*χ*^2^ *P* value < 0.05 or *I*^2^ > 50%). In addition, one-way sensitivity analyses were performed to estimate the effects of studies on the combined results for outcomes if obvious heterogeneity was detected. Otherwise, publication bias was evaluated visually by funnel plots via Review Manager version 5.4.

## Results

### Literature search and study characteristics

**Fig. 1:**
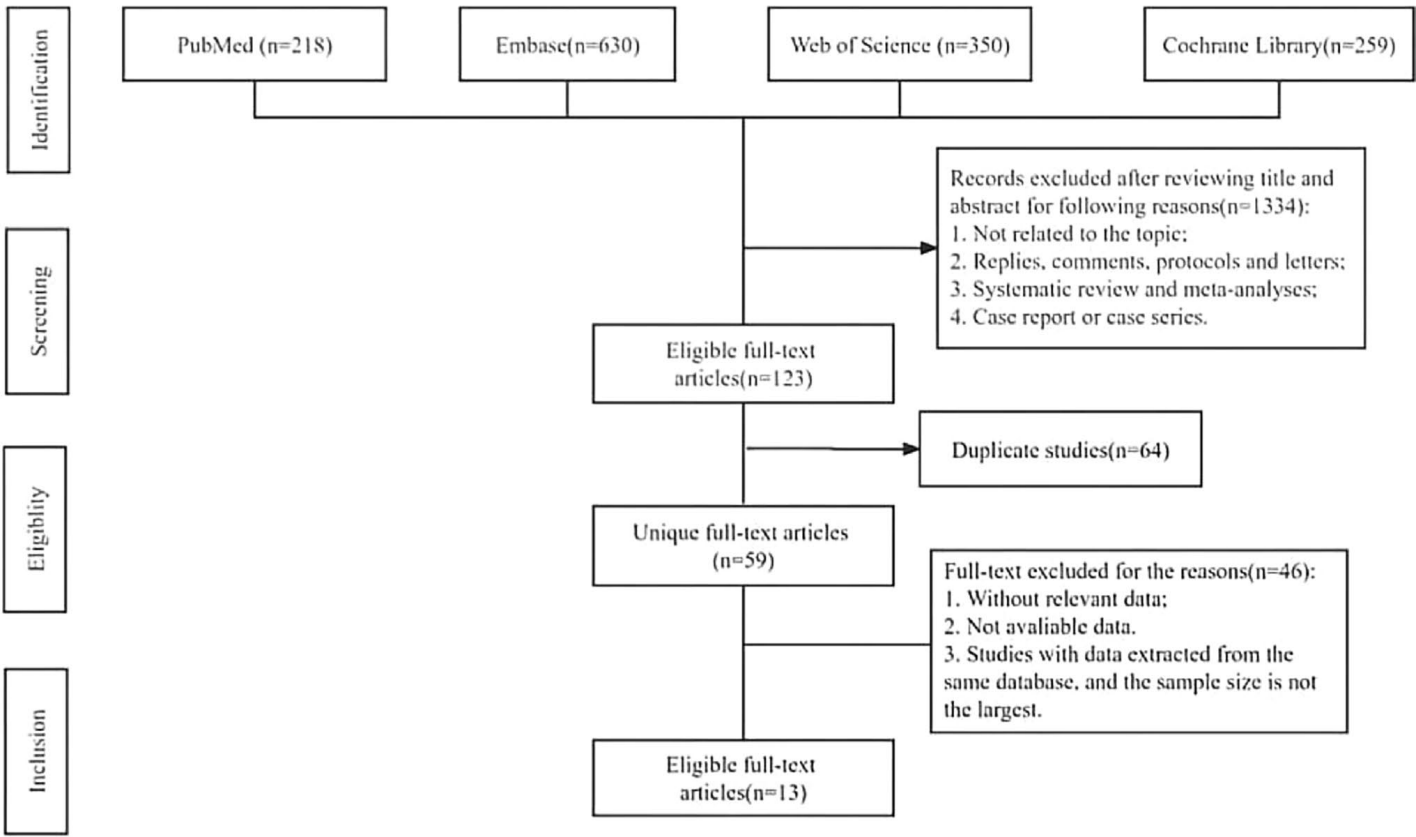
Flowchart of literature selection process

The flowchart of our search and selection process is presented in Fig. 1. In total, 1450 relevant articles in PubMed (*n* = 218), Embase (*n* = 630), Web of Science (*n* = 350) and Cochrane Library (*n* = 259) were identified through a systematic automatic and artificial search. After reviewing the titles and abstracts, 1, 334 articles were excluded, and 59 studies were selected for the second screening after additionally excluding 64 duplicated studies. Finally, 13 full-text articles involving 38,798 patients were included in this meta-analysis [11, 22–33]. Of these studies, 10 were randomized controlled trials (RCTs), and 3 were cohort studies. Patients with sepsis, trauma, and neurological or cardiac situations were recorded separately, and subgroup analysis was performed. Pediatric patients were identified for another subgroup analysis. If studies involved three types of fluids, we repeated the inclusion of the same study with different comparisons.

Table 1 shows the baseline characteristics of the included studies.

**Table 1:** Baseline characteristics of include studies in the meta-analysis

Study	Simple size (*n*)	Country	Study design	Population	Study period	Outcomes			Intervention(s) Compared With 0.9% Saline	Centers	ICU admission			
						Mortality	Renal function	Service utilization			Trauma	Neurosurgery	Cardiac surgery	Sepsis
Raman S, 2023 [22]	516	Australia	RCT	Children	2019–2021	–	AKI	Hospital stay, Length of ICU stay	Gluconate/Acetate–Buffered Solution Lactate-Buffered Solution	1	Yes	Yes	No	Yes
Shephali, 2022 [23]	100	India	RCT	Adults	2019–2020	28-days mortality	–	–	Balanced solution	–	Yes	Yes	No	No
Scioscia, 2022 [24]	1166	USA	Cohort	Children	2017–2019		AKI	Hospital stay, Length of ICU stay	Ringers solution Plasma-Lyte-A	1	No	No	No	No
Yi Bian. 2022 [25]	662	China	Cohort	Adults	2019–2020	30-days	MAKE30, RRT, Final creatinine level≥200% of baseline	Hospital stay, Length of ICU stay, ICU-free days, Ventilator-free days	Bicarbonate Ringer’s Solution	1	Yes	Yes	Yes	Yes
Finfer, 2022 [29]	5037	Australia	RCT	Adults	2017–2020	28, 90-days, hospital	RRT, Maximum creatinine level, Maximum creatinine increase	–	Plasma-Lyte-A	53	Yes	Yes	Yes	Yes
Tseng, 2021 [26]	938	Taiwan,China	Cohort	Adults	–	30, 60, 90-days	–	Hospital stay, Length of ICU stay	Ringer’s Solution	1	No	No	No	Yes
Zampier, 2021 [32]	11, 052	Brazil	RCT	Adults	2017–2020	90-days, hospital mortality	AKI, RRT, Final creatinine level≥200% of baseline	Hospital stay, Length of ICU stay, Ventilator-free days	Balanced solution	75	Yes	No	Yes	Yes
Trepatchayakorn. 2021 [27]	42	Thailand	RCT	Children	2016–2019	Hospital	MAKE30	Hospital stay, Length of ICU stay	Sterofundin, Ringers Solution	1	No	No	No	Yes
Williams, 2020 [30]	66	India	RCT	Children	2017–2018	Hospital	AKI,RRT	Hospital stay, Length of ICU stay	Plasma-Lyte-A	1	No	No	No	No
Semler, 2018 [11]	15, 802	USA	RCT	Adults	2015–2017	30, 60-days	MAKE30, RRT, Final creatinine level≥200% ofbaseline,AKI,Maximum creatinine level, Maximum creatinine increase	ICU-free days, Ventilator-free days	Ringers solution, Plasma-Lyte-A	5	Yes	Yes	Yes	Yes
Ratanarat. 2017 [31]	181	Thailand	RCT	Adults	–	–	AKI, RRT	–	Sterofundin	1	–	–	–	–
Semler, 2017 [28]	974	USA	RCT	Adults	2015	30, 60-days	MAKE30, RRT, Final creatinine level≥200% ofbaseline ,AKI, Maximum creatinine level, Maximum creatinine increase	ICU-free days, Ventilator-free days	Balanced solution	1	No	No	No	Yes
Young, 2015 [33]	2262	New Zealand	RCT	Adults	2014	Hospital mortality	AKI, RRT	Hospital stay, Length of ICU stay	Plasma-Lyte-A	4	Yes	Yes	Yes	Yes

*RCT* Randomized controlled trial

### Mortality outcomes

**Fig. 2:**
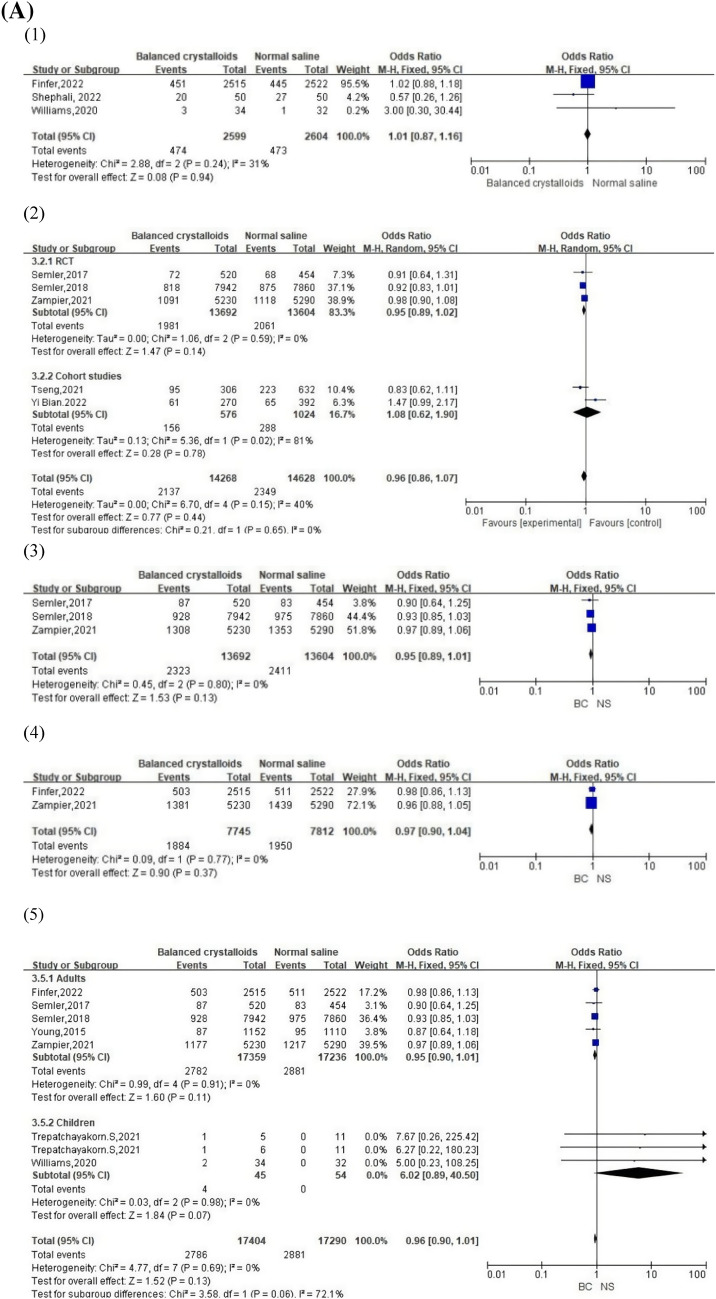

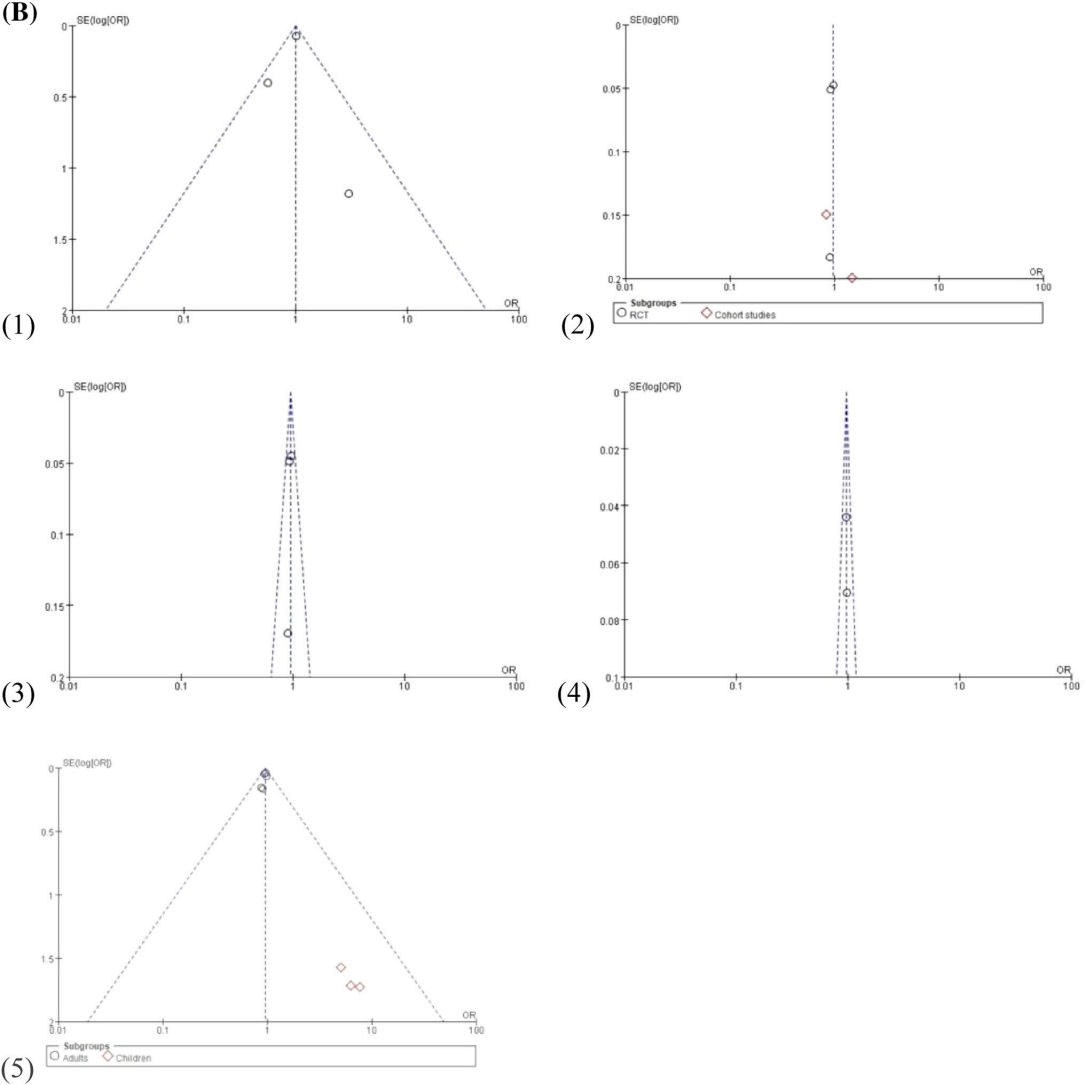
**A** Forest plots of mortality outcomes: (1) 28-day mortality, (2) 30-day mortality, (3) 60-day mortality, (4) 90-day mortality, and (5) hospital mortality. **B** Funnel plot of mortality outcomes: (1) 28-day mortality, (2) 30-day mortality, (3) 60-day mortality, (4) 90-day mortality, and (5) hospital mortality

Data on mortality outcomes were synthesized from 10 studies. Subgroup analysis was performed according to mortality time. Pooled analysis revealed that each patient subgroup had no significant difference in mortality outcomes based on the use of balanced crystalloid solutions and normal saline, with no apparent heterogeneity, aside from the 30-day mortality group in cohort studies (Fig. 2A). A visual assessment of the funnel plot indicated apparent publication bias in 28-day mortality and hospital mortality groups (Fig. 2B).

### Renal function outcomes

#### MAKE30

MAKE30 was described as the composite of death, new receipt of RRT, or persistent renal dysfunction within 30 days after enrollment. In total, 4 studies (3 RCTs and 1 cohort study) reported the data of MAKE30 for 3,709 patients (1,776 balanced crystalloid group vs. 1,933 normal saline group). After excluding the data from the cohort study, no significant difference was detected between the two groups (OR, 0.92; 95% CI, [0.85, 1.00]; *p* = 0.06] with no obvious heterogeneity (*I*^2^ = 0%, *p* = 0.53), indicating that the MAKE30 in the balanced crystalloid solutions group was similar to that in the normal saline group. However, the funnel plot indicated publication bias in the primary studies.

#### AKI

In total, 9 studies involving 3,092 patients (1512 balanced crystalloid group vs. 1,580 normal saline group) were included in the meta-analysis. After grouping studies according to patient age, our results demonstrated that adult patients who accepted balanced crystalloid solutions had a lower total AKI incidence than those who accepted saline (OR, 0.92; 95% CI, [0.86, 1.00]; *p* = 0.04). However, in children patients and adults with KDIGO ≥ 2, both groups had similar incidences in AKI (*children*: OR, 1.26; 95% CI, [0.70, 2.25]; *p* = 0.44; *adults with KDIGO* ≥ *2*: OR, 0.94; 95% CI, [0.88, 1.01]; *p* = 0.09) with no significant heterogeneity (*adults and children*: *I*^2^ = 0%). In addition, a visual assessment of the funnel plot indicated slight publication bias among the pediatric patients.

#### RRT

Of the 8 studies reporting RRT, 6 were selected for analysis. Pooled analysis detected no significant difference between the balanced crystalloid and saline in the RRT rate (OR, 0.94; 95% CI, [0.88, 1.01]; *p* = 0.09), with no significant heterogeneity (*I*^2^ = 26%, *p* = 0.24). In addition, funnel plots revealed no apparent publication bias. After developing subgroups according to ICU admission cause (sepsis), we still reach the same conclusion with significant heterogeneity (Figure S1).

#### Final creatinine level ≥ 200% of baseline

In total, 3 studies reported 4,095 patients (2,015 balanced crystalloid group vs. 2,080 normal saline group) whose final creatinine levels were double that of the baseline. Similar results were observed between these two groups in our analysis (OR, 0.96; 95% CI, [0.90, 1.03]; *p* = 0.26), with no statistically significant heterogeneity (*I*^2^ = 0%, *p* = 0.60). The funnel plot did not suggest a publication bias.

#### Maximum creatinine increasing and maximum creatinine level

Serum creatinine has been considered as a gold standard determiner for the diagnosis of AKI for approximately 100 years [34]. Creatinine levels are strongly associated with the glomerular filtration rate. Hence, an increase of maximum creatinine increasing may represent the degree of kidney impairment. Data on the maximum creatinine increasing and maximum level after intravenous fluid therapy were extracted from 3 studies with 21,813 patients in the ICU (10,977 balanced crystalloid group vs. 10,836 normal saline group). No significant difference and heterogeneity were detected from the forest plot between balanced crystalloid solutions and 0.9% normal saline (maximum creatinine increasing: WMD, -0.00; 95% CI, [-0.01, 0.01]; *p* = 0.53 and *I*^2^ = 0%; *p* = 0.99; maximum creatinine level: WMD, 0.00; 95% CI, [-0.02, 0.02]; *p* = 0.85 and *I*^2^ = 0%; *p* = 0.44). In addition, no significant publication bias was observed from the funnel plot.

**Fig. 3:**
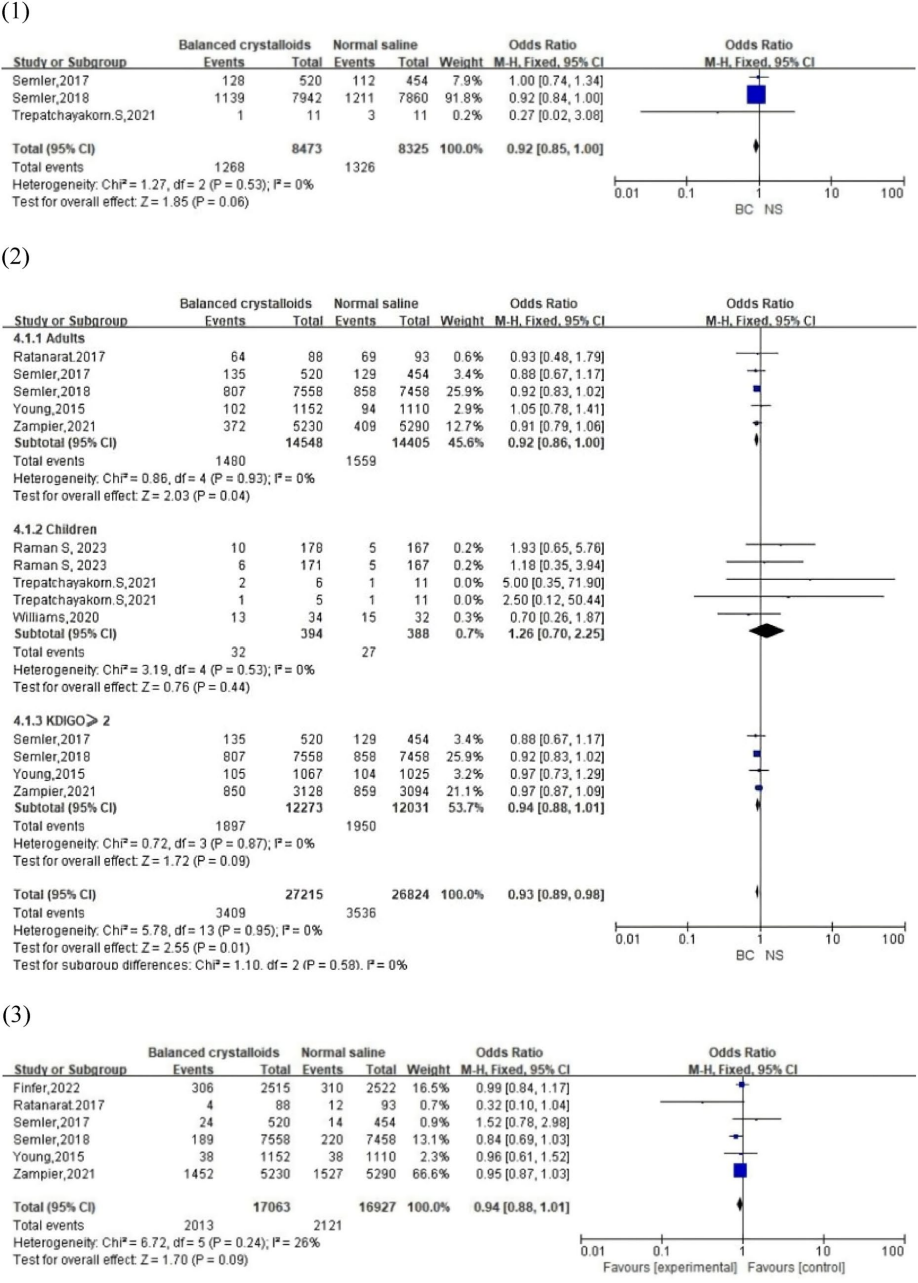

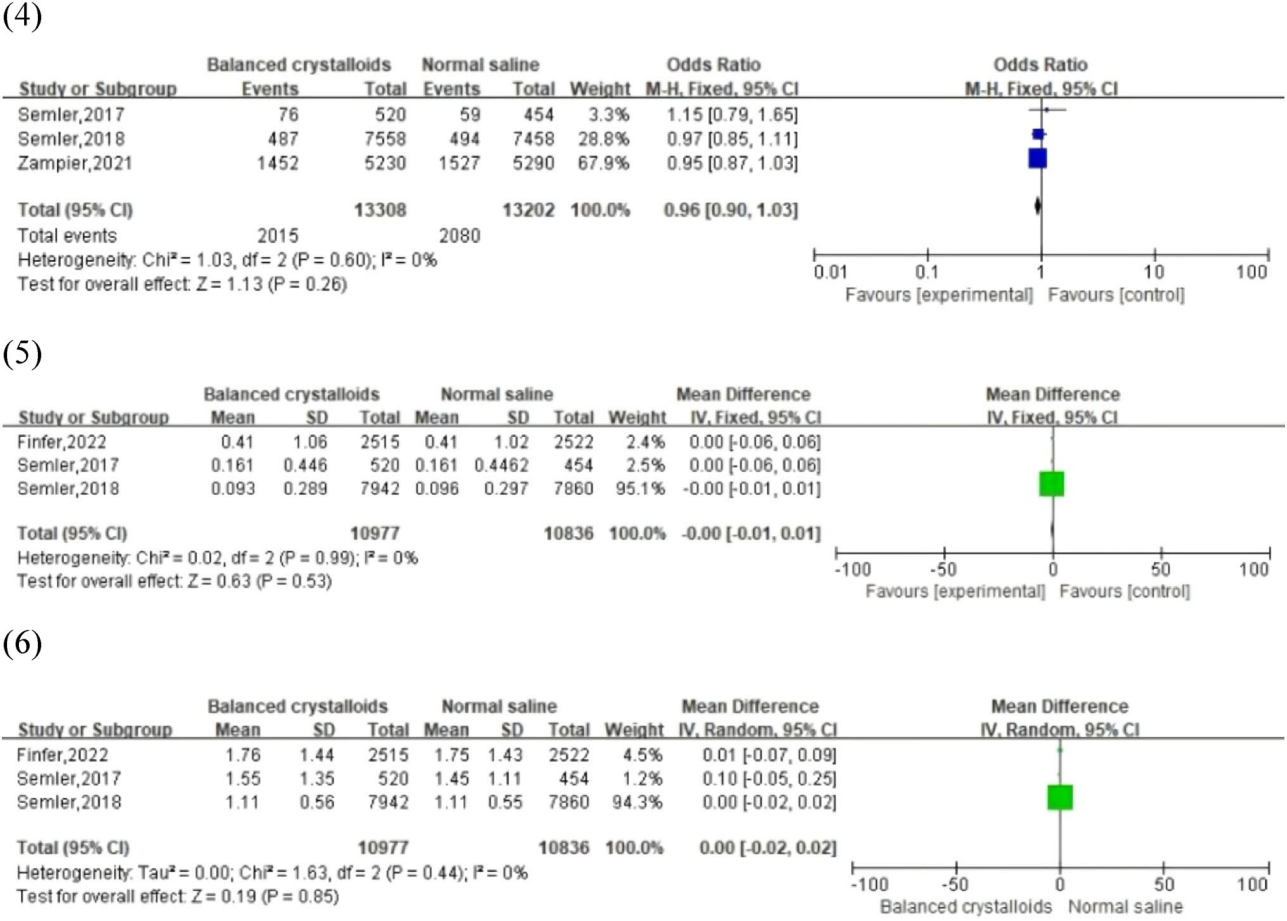
Forest plots of renal-related outcomes: (1) MAKE30; (2) AKI; (3) RRT; (4) final creatinine level ≥ 200% of baseline; (5) maximum creatinine increasing; (6) maximum creatinine level

**Fig. 4:**
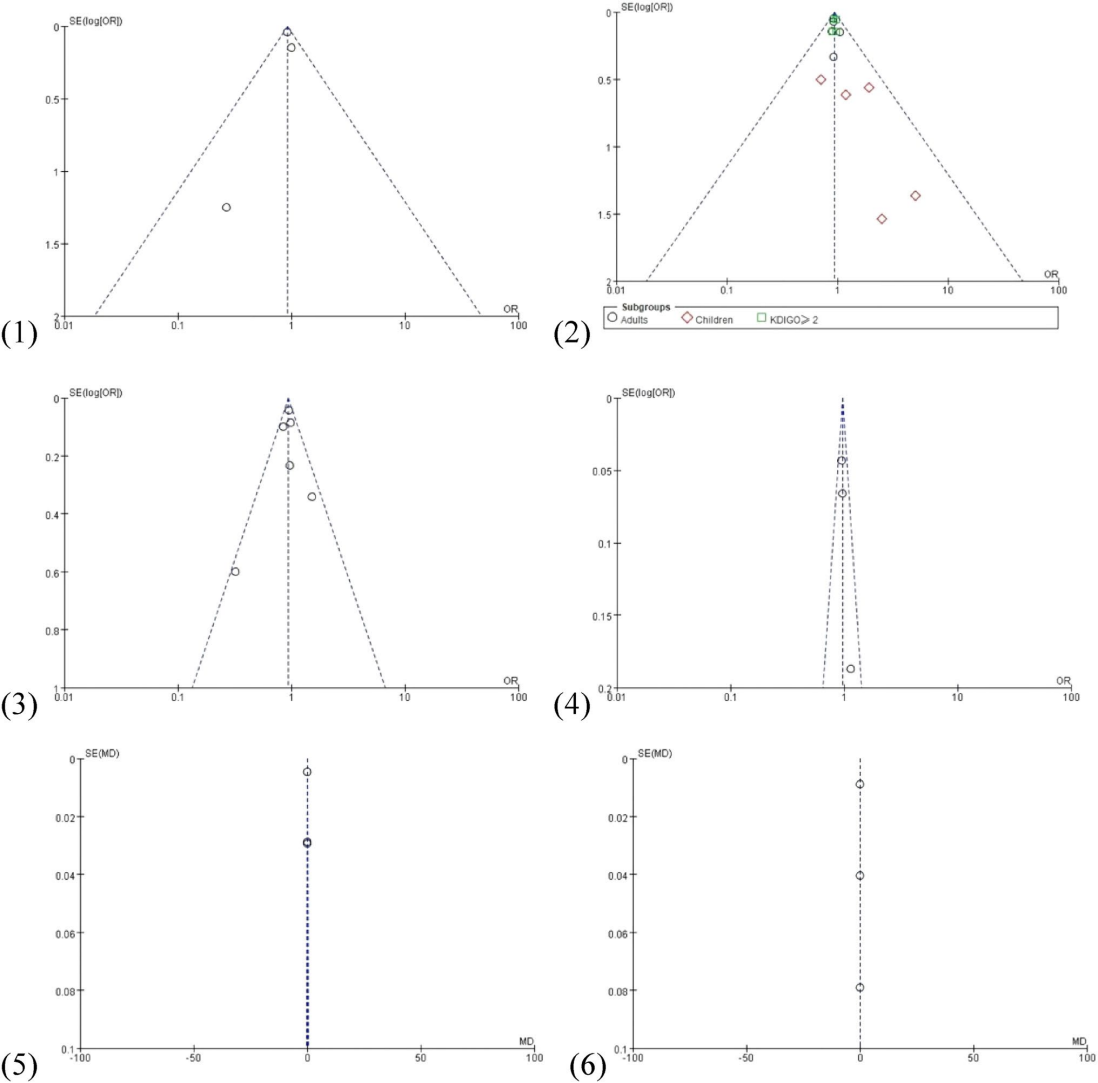
Funnel plot of mortality outcomes: (1) MAKE30; (2) AKI; (3) RRT; (4) final creatinine level ≥ 200% of baseline; (5) maximum creatinine increasing; (6) maximum creatinine level

All analyses and forest plots of renal-related outcomes are shown in Fig. 3. Figure 4 presents the funnel plots of the above outcomes.

### Service utilization outcomes

#### Length of ICU stay

In total, 8 studies with 15,960 involved patients (7872 balanced crystalloid group vs. 8088 normal saline group) revealed the length of ICU stay data. After conducting subgroup analyses, our study reported that the adults who accepted balanced crystalloids solution had longer lengths of ICU stay with no significant heterogeneity (*adults*: WMD, 0.02; 95% CI, [0.01, 0.03]; *p* = 0.0004 and *I*^2^ = 0%; *p* = 0.96), while no difference was found in both two groups of pediatric patients (*children*: WMD, – 0.05; 95% CI, [– 0.48, 0.39]; *p* = 0.84 and *I*^2^ = 68%; *p* = 0.02). In addition, for both RCT and cohort studies, the two groups had similar lengths of ICU stay (*RCT*: WMD, – 0.01; 95% CI, [– 0.13, 0.11]; *p* = 0.85; *cohort studies*: WMD, – 0.94; 95% CI, [– 4.71, 2.83]; *p* = 0.63), with apparent heterogeneity in the cohort group (*RCT*: *I*^2^ = 48%, *p* = 0.06; *cohort studies*: *I*^2^ = 99%, *p* < 0.00001). Funnel plots revealed a slight publication bias in the pediatric group.

#### Length of hospital stay

In total, 7 articles reported the length of hospital stay. The results of the pediatric subgroup indicated patients who accepted balanced crystalloid solutions had shorter hospital stay time compared with those in normal saline group, with no obvious heterogeneity (WMD, – 1.10; 95% CI, [– 2.10, – 0.10]; *p* = 0.03 and *I*^2^ = 17%; *p* = 0.30). In other subgroups, including the RCT group, cohort group, and adult group, different degrees of heterogeneity were revealed clearly with similar hospital lengths of stay (*adults*: WMD, − 0.05; 95% CI, [− 0.45, 0.35]; *p* = 0.85 and *I*^2^ = 80%; *p* = 0.03; *RCT*: WMD, − 0.35; 95% CI, [− 0.86, 0.15]; *p* = 0.17 and *I*^2^ = 65%; *p* = 0.009; *cohort studies*: WMD, − 2.48; 95% CI, [− 6.27, 1.31]; *p* = 0.20 and *I*^2^ = 89%; *p* = 0.002). Funnel plots also revealed apparent publication bias in pediatric and RCT groups.

#### ICU-free days

Data on ICU-free days were reported in 2 RCT studies that involved 16,776 patients (8,462 in balanced crystalloids and 8,314 in saline) totally. The forest plot revealed that these two groups had similar ICU-free days with no significant heterogeneity (WMD = 0.00, 95% CI = [– 0.07, 0.07], *p* = 1.00, *I*^2^ = 0%, *p* = 1.00). In addition, the funnel plot also detected a negative publication bias.

#### Ventilator-free days

Further, 3 articles reported ventilator-free days of 13,679 patients in the balanced crystalloid group and 13,601 in the normal saline group (27,280 in total). Our study reports that there was no significant difference between those two groups with significant heterogeneity (WMD, 0.41; 95% CI, [− 0.06, 0.88]; *p* = 0.09 and *I*^2^ = 92%; *p* < 0.00001). Publication bias was not apparent according to the results of the funnel plot.

**Fig. 5:**
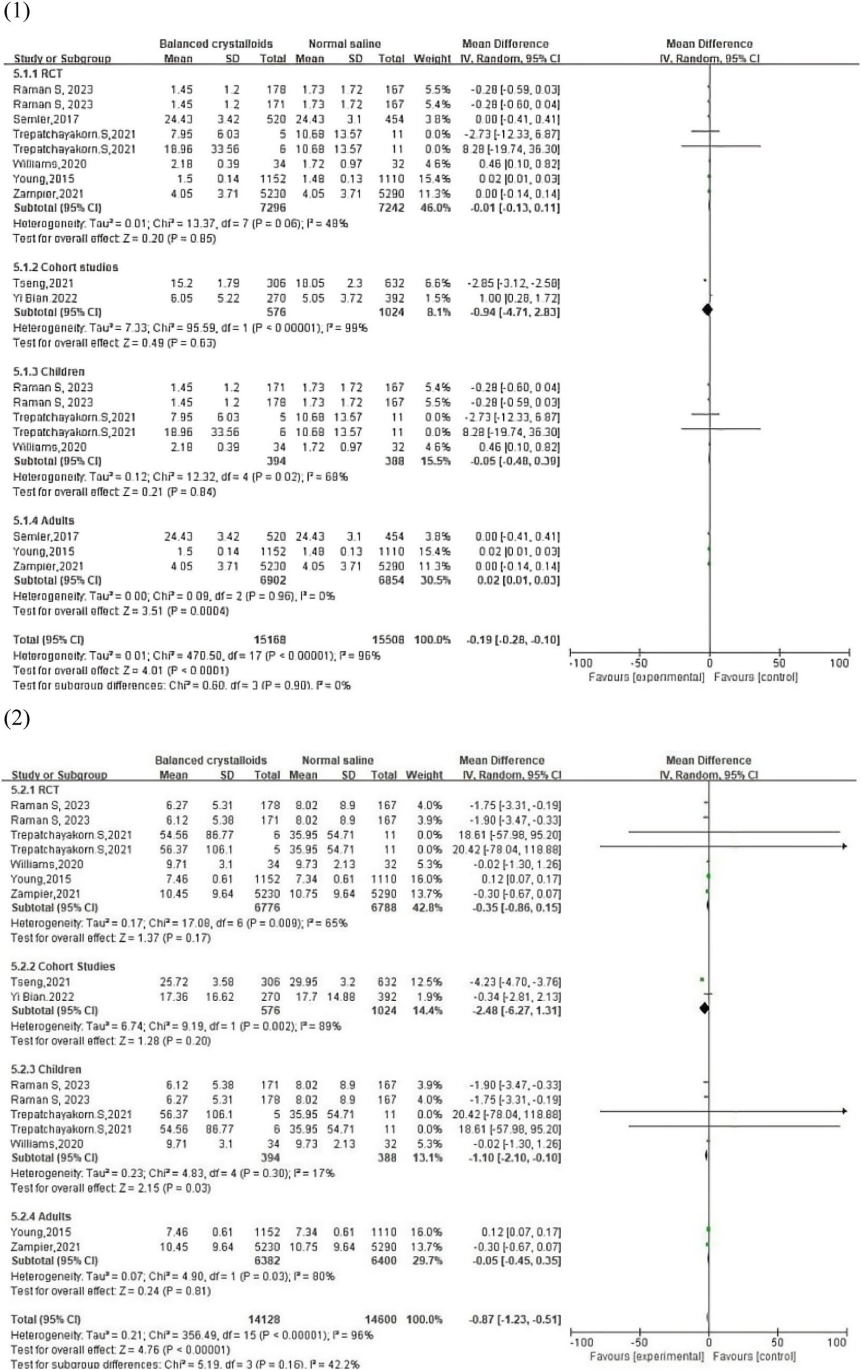

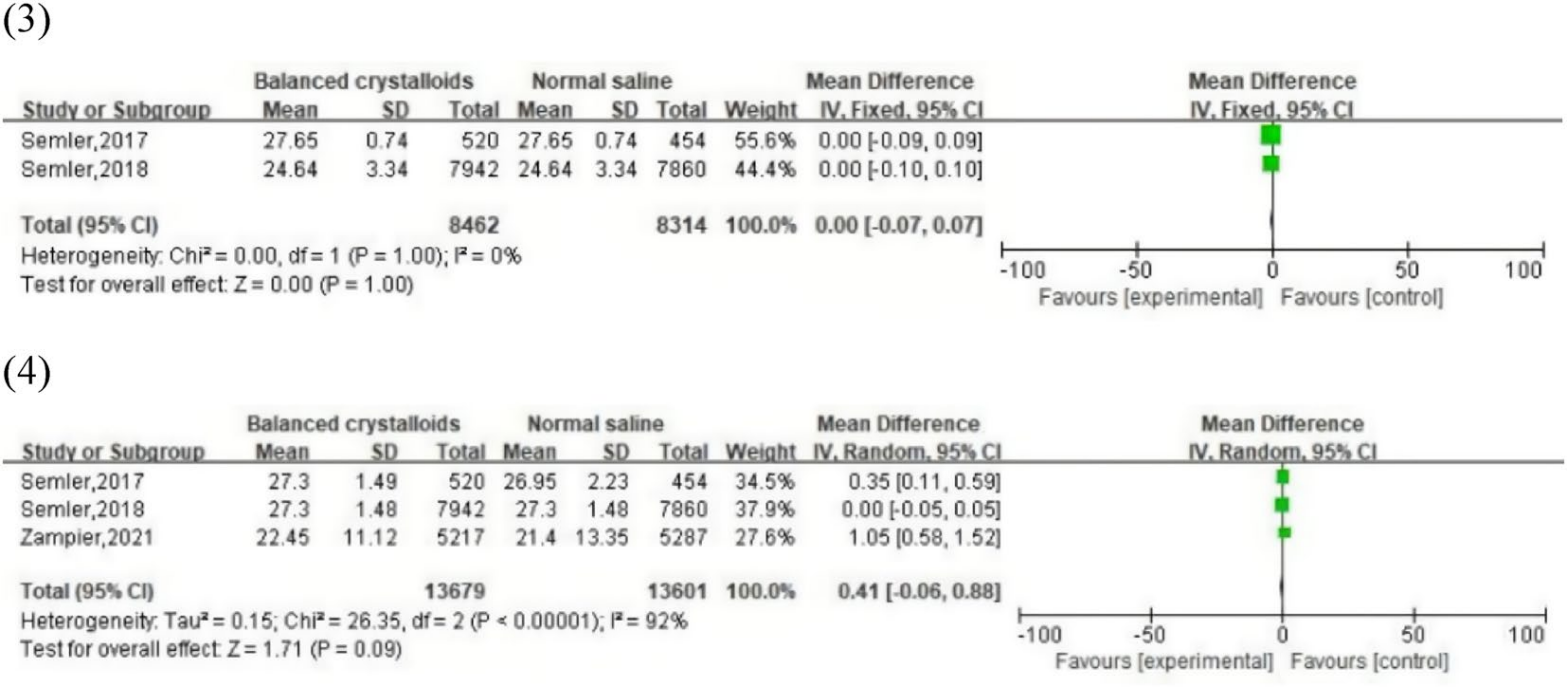
Forest plots of service utilization outcomes: (1) length of ICU stay; (2) length of Hospital stay; (3) ICU-free days; (4) ventilator-free days

**Fig. 6:**
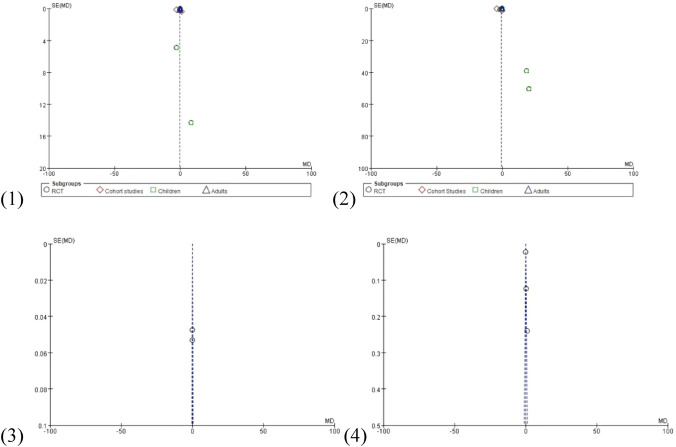
Funnel plot of service utilization outcomes: (1) length of ICU stay; (2) length of hospital stay; (3) ICU-free days; (4) ventilator-free days

Related information of service utilization outcomes is shown in Figs. 5 and 6.

### Sensitivity analysis

Sensitivity analysis was conducted to resolve the existence of heterogeneity in outcomes of ventilator-free days by excluding studies in the series through Review Manager 5.4. Results revealed that significant heterogeneity could not be eliminated by excluding any involved primary studies. Moreover, all analyses in subgroups of cohort studies report apparent heterogeneity that could not be resolved by removing any study.

## Discussion

As the cornerstone treatment of critically ill patients, intravenous fluid administration is necessary for management in ICUs [35, 36]. Normal saline is the earliest and most common crystalloid solution that has been widely used in fluid resuscitation, while balanced crystalloid solutions have a different composition, as chloride anions are replaced with bicarbonate or buffers [37]. To maintain the homeostasis of patients, balanced crystalloid solutions are classified based on their elements and have different indications, contraindications, and prognoses. The balanced crystalloids used in our study mainly include Plasma-Lyte-A, Sterofundin, sodium bicarbonate Ringer’s solution, and lactated Ringer’s solution. The safety of organ function with intravenous fluids among patients in the ICU is prioritized by monitoring arterial blood gas. The influence of a high chloride concentration in saline on acidosis has long been reported in existing literature [38, 39], and its specific mechanisms may be related to plasma dilution and changes in blood carbonic acid concentrations [40, 41]. One retrospective cohort study involving 60,374 patients divided intravenous fluids into three groups (saline, saline with crystalloid, and saline with colloform) and investigated the mortality of each group. The study found that the saline group and the balanced crystalloid group had the highest and lowest mortality, rates, respectively [42]. However, the severity of acidosis and its impact on patients’ internal environments and organ functions remain uncertain. Some studies suggests that as patients can develop a strong tolerance to acidosis [43, 44], it cannot be the definitive cause of kidney dysfunction [45]. In addition, although several studies have reported that normal saline can reduce the glomerular filtration rate and renal artery flow velocity [12], these adverse effects can be reduced through comprehensive treatments including vasopressor drugs, respiratory stimulant drugs, or mechanical ventilation in the ICU. Moreover, balanced crystalloid solution can potentially be unsafe. For example, Plasma-Lyte-A can increase the risk of hyperkalemia and hypermagnesemia in patients.

Our study focused on the following three important indicators of prognosis: mortality, renal-related, and service utilization outcomes. The mortality outcomes are divided into several subgroups including 28-day, 30-day, 60-day, 90-day, and hospital mortality. Some previous studies reported that saline administration can increase the mortality of critically ill patients [7, 11], while other studies report no significant improvement in mortality after administration of balanced crystalloid solutions [25, 29]. Our analysis found that the mortality rate was similar after administration of balanced crystalloid solutions and normal saline in critically ill patients and further found that balanced crystalloid solutions did not have an advantage in reducing mortality in ICU patients, both children and adults. However, by contrast, a recent meta-analysis involving 13 RCTs showed that balanced crystalloid solutions can reduce mortality in critically ill adults. Our analysis concluded that there was no evident difference in MAKE30 between these two groups, similar to previous studies [46, 47]. It was detected that the balanced crystalloid solutions could not decrease the MAKE30 rate of ICU patients. However, publication bias was present in our study, as we combined adult and pediatric patients, suggesting that additional research is necessary to reduce publication bias. In addition, for other renal-related outcomes, our analysis found that all outcomes, except AKI, between balanced crystalloid solutions and normal saline had similar results. We revealed that balanced crystalloid solutions could reduce AKI incidence among adults, but not among children. This result resembles that of a recent study that reported relative outcomes among adults in critical conditions and further verified the previous confirmations. Furthermore, regarding service utilization outcomes, length of ICU and hospital stay, ICU-free days, and ventilator-free days were also included in our analysis. Primary analysis showed that although length of ICU stay among all patients was similar between the two groups, the subgroup of both adults and children revealed that balanced crystalloid solution yielded a longer ICU stay than saline, different from previous studies. No significant difference between balanced crystalloid solution and normal saline was present in other service utilization outcomes.

Moreover, our study found similar RRT rates among adult patients in both groups, although the risk of AKI was lower in patients treated with balanced crystalloid solutions. The AKI Network (AKIN) criteria classify AKI into risk, injury, and failure categories [48, 49]. In general, only patients with end-stage renal failure need RRT, and it is unnecessary for patients classified under AKIN Stage 1 or 2. However, although we had conducted subgroup analysis according to KDIGO criteria, we still could not include more detailed subgroups based on the AKIN classification because of the limitation of data in primary studies. Hence, more analyses, including the correlations between categories of AKI and different intravenous fluid infusion therapy are needed.

Our study further contributes to the understanding of the efficacy of balanced crystalloid solution and normal saline. Compared with previous studies, our results included as many indicators as possible to develop an overall assessment of the two groups. To reduce the bias caused by age, both adults and children were involved in our analysis. Subgroups were also created based on the type of study and age. In addition, the causes of ICU admission among patients were also recorded because some causes have different influences on the volume of infusion. However, we also acknowledge that there are several limitations to our study. For example, special situations like sepsis or brain trauma need more intravenous fluids to resuscitate, but we did not develop additional subgroups to analyze patients with different admission causes to compensate for the unavailability of data in primary studies, which may impact the final result with discrepancy of infusion volumes. Further, due to the small size of primary studies, the types of balanced crystalloid solutions were not allocated to subgroups, creating potential bias of our results, especially for renal-related outcomes. Moreover, some outcomes in our analysis, like all outcomes of cohort studies, have significant heterogeneity that cannot be eliminated, decreasing the reliability of our results. Thus, although our analysis revealed some meaningful results comparing balanced crystalloid solutions and normal saline, more primary studies, especially surrounding fluids therapy among children in the ICU are still needed in the future.

## Conclusion

Compared with saline, balanced crystalloid solutions could not reduce the risk of mortality and renal-related outcomes, including MAKE30, RRT, maximum creatinine increasing, maximum creatinine level, and final creatinine level ≥ 200% of baseline, but could reduce total AKI incidence in adults. Regarding service utilization outcomes, balanced crystalloid solutions are associated with a longer length of ICU stay among adult patients and a shorter length of hospital stay among pediatric patients than saline. To address existing limitations, more high-quality studies are necessary in the future.

### Supplementary Information

Below is the link to the electronic supplementary material.

Supplementary file1 (PDF 680 KB)

Supplementary file2 (DOC 16 KB)

Supplementary file3 (DOCX 17 KB)

## Data Availability

The authors confirm that the data supporting the findings of this study are available within the article and its supplementary materials.
