# Supplementary material for: Balanced crystalloid solutions versus normal saline in intensive care units: a systematic review and meta-analysis
Source: Int Urol Nephrol. 2023 Apr 5;55(11):2829–44. doi: 10.1007/s11255-023-03570-9 (PMC10560196; doi:10.1007/s11255-023-03570-9)
Supplement: Supplementary file 2 — Supplementary file2 (DOC 16 KB) [file 11255_2023_3570_MOESM2_ESM.doc]

#1=(((((((((((((((((((((((("Ringer's Lactate") OR (Ringer's Lactate)) OR (Ringer's solution)) OR (lactated Ringer’s solution)) OR (Lactate, Ringer's)) OR (Lactated Ringers Solution)) OR (Ringers Solution，Lactated)) OR (Ringers Lactate)) OR (Lactate, Ringers)) OR (Lactated Ringer Solution)) OR (Ringer's Solution, Lactated)) OR (Balanced multielectrolyte Solution)) OR (Balanced crystalloids solution)) OR (multielectrolyte)) OR (crystalloids solution)) OR (multielectrolyte Solution)) OR (Balanced Crystalloids)) OR (Balanced)) OR (Hanks' Balanced Salt Solutions)) OR (Hanks Balanced Solutions)) OR (Hanks Solutions) ) OR (Hartmanns Solution) ) OR (Plasma-Lyte)) OR (Plasmalyte)) OR (crystalloid solution) AND ((ffrft[Filter]) AND (fft[Filter]))

#2=((((((("Saline Solution") OR (Saline Solution)) OR (0.9% Saline)) OR (Saline)) OR (Saline, 0.9%)) OR (0.9% NaCl)) OR (Normal Saline)) OR (Saline, Normal) AND ((ffrft[Filter]) AND (fft[Filter]))

#3=(((((((((((((((((("Sepsis") OR (sepsis)) OR (pyemia)) OR (pyohemia)) OR (septicemia)) OR (poisoning, blood)) OR (blood poisoning)) OR (toxic shock)) OR (Shock, Toxic)) OR (septic shock)) OR (Shock, Septic)) OR (endotoxic shock)) OR (Shock, endotoxic)) OR ("Intensive Care Units")) OR (Intensive Care Units)) OR (Care Unit, Intensive)) OR (Care Units, Intensive)) OR (Intensive Care Unit)) OR ("Intensive Care Unit") AND ((ffrft[Filter]) AND (fft[Filter]))

#1 AND #2 AND #3

#1=((((((((((((((((((((((((TS=("Ringer's Lactate")) OR TS=(Ringer's Lactate)) OR TS=(Ringer's solution)) OR TS=(lactated Ringer’s solution)) OR TS=(Lactate, Ringer's)) OR TS=(Lactated Ringers Solution)) OR TS=(Ringers Solution，Lactated)) OR TS=(Ringers Lactate)) OR TS=(Lactate, Ringers)) OR TS=(Lactated Ringer Solution)) OR TS=(Ringer's Solution, Lactated)) OR TS=(Balanced multielectrolyte Solution)) OR TS=(Balanced crystalloids solution)) OR TS=(multielectrolyte)) OR TS=(crystalloids solution)) OR TS=(multielectrolyte Solution)) OR TS=(Balanced Crystalloids)) OR TS=(Balanced)) OR TS=(Hanks' Balanced Salt Solutions)) OR TS=(Hanks Balanced Solutions)) OR TS=(Hanks Solutions)) OR TS=(Hartmanns Solution)) OR TS=(Plasma-Lyte)) OR TS=(Plasmalyte)) OR TS=(crystalloid solution)

#2=(((((((TS=(Saline Solution)) OR TS=(Saline Solution)) OR TS=(0.9% Saline)) OR TS=(Saline)) OR TS=(Saline, 0.9%)) OR TS=(0.9% NaCl)) OR TS=(Normal Saline)) OR TS=(Saline, Normal)

#3=((((((((((((((((((TS=(Sepsis)) OR TS=("Sepsis")) OR TS=(pyemia)) OR TS=(pyohemia)) OR TS=(septicemia)) OR TS=(poisoning, blood)) OR TS=(blood poisoning)) OR TS=(toxic shock)) OR TS=(Shock, Toxic)) OR TS=(septic shock)) OR TS=(Shock, Septic)) OR TS=(endotoxic shock)) OR TS=(Shock, endotoxic)) OR TS=("Intensive Care Units")) OR TS=(Intensive Care Units)) OR TS=(Care Unit, Intensive)) OR TS=(Care Units, Intensive)) OR TS=(Intensive Care Unit)) OR TS=("Intensive Care Unit")

#1 AND #2 AND #3 WITH OPEN ACCESS
