# Supplementary material for: Balanced crystalloid solutions versus normal saline in intensive care units: a systematic review and meta-analysis
Source: Int Urol Nephrol. 2023 Apr 5;55(11):2829–44. doi: 10.1007/s11255-023-03570-9 (PMC10560196; doi:10.1007/s11255-023-03570-9)
Supplement: Supplementary file 3 — Supplementary file3 (DOCX 17 KB) [file 11255_2023_3570_MOESM3_ESM.docx]

| Supplement Table 3. Risk of Bias Assessment. | | | | | | | | |
| --- | --- | --- | --- | --- | --- | --- | --- | --- |
| ***Risk of Bias Assessment for Prospective Trials (Cochrane Collaboration Tool)*** | | | | | | | | |
| **Bias** | **Selection** | | **Performance** | **Detection** | **Attrition** | **Reporting** | **Other** | |
| **Trial** | **Random Sequence Generation** | **Allocation Concealment** | **Blinding of Participants and Personnel** | **Blinding of Outcome Assessment** | **Incomplete Outcome Data** | **Selective Reporting** | **Other Bias** | |
| Raman S, 2023 | + | + | + | + | + | + | + | |
| Shephali, 2022 | + | + | ？ | + | + | + | + | |
| Finfer,2022 | + | + | + | + | + | + | + | |
| Zampier,2021 | + | + | + | + | ？ | + | + | |
| Trepatchayakorn.S,2021 | + | + | + | + | + | + | + | |
| Williams,2020 | + | + | ？ | + | + | + | + | |
| Semler,2018 | ？ | ？ | − | + | + | + | + | |
| Ratanarat.2017 | ？ | ？ | ？ | ？ | + | ？ | − | |
| Semler,2017 | ？ | ？ | − | + | + | + | + | |
| Young,2015 | + | + | + | + | + | + | + | |
| ***Risk of Bias Assessment for Cohort Studies (Newcastle Ottawa Tool)*** | | | | | | | | |
| **Study** | **Representativeness of the Exposed Cohort** | **Selection of the Nonexposed Cohort** | **Ascertainment of Exposure** | **Demonstration That the Outcome of Interest Was Not Present at Start of Study** | **Comparability of Cohorts on the Basis of the Design or Analysis** | **Assessment of Outcome** | **Was Follow-up Long Enough for Outcomes to Occur** | **Adequacy of Follow-up Cohorts** |
| **Bias** | **Selection** | | | | **Comparability** | **Outcome** | | |
| Scioscia A, 2022 | * | * | * | * | ** | * | * | |
| Yi, 2022 | * | * | * | * | ** | * | * | |
| Tseng, 2021 | * | * | * | * | ** | * | * | |
| a: +: Low risk of bias. −: High risk of bias. ?: Unclear risk of bias. *Category is addressed adequately. **Comparability category is address adequately. | | | | | | | | |
